# Heterozygosity of the Complex Corfu δ^0^β^+^ Thalassemic Allele (*HBD* Deletion and HBB:c.92+5G>A) Revisited

**DOI:** 10.3390/biology11030432

**Published:** 2022-03-11

**Authors:** Christos Kattamis, Myrto Skafida, Polyxeni Delaporta, Christina Vrettou, Joanne Traeger-Synodinos, Christalena Sofocleous, Antonis Kattamis

**Affiliations:** 1 Thalassemia Unit, Division Pediatric Hematology-Oncology, First Department of Pediatrics, National & Kapodistrian University of Athens, “Aghia Sophia” Children’s Hospital, 11527 Athens, Greece; ckatamis@med.uoa.gr (C.K.); myrto.skafida@downstate.edu (M.S.); polyxenidelaporta@yahoo.gr (P.D.); 2 Laboratory of Medical Genetics, National & Kapodistrian University of Athens, “Aghia Sophia” Children’s Hospital, 11527 Athens, Greece; cvrettou@med.uoa.gr (C.V.); jtraeger@med.uoa.gr (J.T.-S.)

**Keywords:** Corfu δ^0^β^+^ thalassemic allele, β-thalassemia variants, β-thal hematological phenotype, normal HbA2, high HbF

## Abstract

**Simple Summary:**

The Corfu δ^0^β^+^ thalassemic allele, a unique thalassemic allele combining a deletion of the δ-globin (*HBD*) and a single nucleotide variant in the β-globin gene (*HBB*) in cis has, so far, been described only in individuals of Greek origin. The heterozygosity of Corfu δ^0^β^+^ is detected in 1–2% of the β-thalassemia carrier population and presents a distinct hematological phenotype of microcytic, hypochromic anemia with normal HbA_2_ and elevated HbF levels. The study of the Corfu δ^0^β^+^ allele is important for genotype resolution, genetic counseling and prenatal/antenatal diagnosis, and the management of patients.

**Abstract:**

The Corfu δ^0^β^+^ thalassemic allele is a unique thalassemic allele consisting of the simultaneous presence in cis of a deletion of the δ-globin (Hemoglobin Subunit Delta, *HBD*) and a single nucleotide variant in the β-globin gene (Hemoglobin Subunit Beta, *HBB*). The allele has, so far, been described in individuals of Greek origin. The objectives of the study are to ascertain the prevalence of the Corfu δ^0^β^+^ allele in comparison to other β-thalassemia variants encountered in Greece using our in-house data repository of 2558 β-thalassemia heterozygotes, and to evaluate the hematological phenotype of Corfu δ^0^β^+^ heterozygotes in comparison to heterozygotes with the most common β^+^- and deletion α^0^- thalassemia variants in Greece. The results of the study showed a relative incidence of heterozygotes with Corfu δ^0^β^+^ at 1.56% of all β-thalassemic alleles, and a distinct hematological phenotype of the heterozygotes characterized by microcytic, hypochromic anemia with normal levels of HbA_2_ (Hemoglobin A2) and elevated HbF (Hemoglobin F) levels. The application of a specific methodology for the identification of the Corfu δ^0^β^+^ allele is important for precise prenatal and antenatal diagnosis programs in Greece.

## 1. Introduction

δ-thalassemia is caused by defects in the δ-globin (Hemoglobin Subunit Delta, *HBD*) gene that result in lower HbA_2_ levels. Isolated δ-thalassemia has no clinical significance but may confound the diagnosis of individuals with β-thalassemia. δβ-thalassemia is a genetically heterogeneous group of disorders in which both the expression of the δ-globin gene and the in cis β-globin (Hemoglobin Subunit Beta, *HBB*) gene are affected [1,2].

The unique complex Corfu δ^0^β^+^ allele was first described in one of our patients from the Greek island of Corfu, who presented with non-transfusion dependent thalassemia at the age of 4 years with hemoglobin (Hb) of 9.2g/dL, comprising mainly HbF and low levels of HbA (5.8% with zero HbA_2_). The parents of the propositus were characterized as heterozygotes for “normal HbA_2_, type 2 thalassemia”, which has a distinct hematological phenotype, with reduced red blood cells indices, decreased osmotic fragility and an unbalanced α/β-globin synthesis ratio comparable to that of heterozygotes with typical β^0^-thalassemia variants distinguished by increased HbA_2_ levels [3,4]. Molecular studies on the propositus identified homozygosity of a complex allele carrying a 7.2 kb deletion δ^0^ variant, partially removing δ-globin gene, in cis to IVSI-5G>A β^+^ variant [HBB:c.92+5G>A; NG_000007.3:g.57237_64443del7207] [4,5]. Further studies from our group had, very early, indicated a link between the Corfu δ^0^β^+^ allele and a statistically significant raised HbF level [6].

Studying naturally occurring thalassemic variants has significantly improved our understanding of the mechanisms underlying the developmental switching of hemoglobin (Hb) during normal growth [7]. Reactivation of the γ-globin genes (Hemoglobin Subunit Gamma1/2, *HBG1/HBG2*) for the treatment of thalassemia and sickle cell disease has recently been the focus of many research efforts, with respective gene-editing clinical trials already showing very promising results [8].

To the best of our knowledge, the Corfu δ^0^β^+^ allele has been recorded exclusively amongst β-thalassemia heterozygotes of Greek origin, whereas limited data on its presentation have been published. This survey addresses: (i) the relative prevalence of the Corfu δ^0^β^+^ allele amongst all β-thalassemia variants prevailing in Greece; (ii) the precise definition hematological phenotype of Corfu δ^0^β^+^ heterozygotes and (iii) the evaluation of the specific hematological phenotype in heterozygotes with the Corfu δ^0^β^+^ allele compared to heterozygotes with either with β^+^ (IVSI-110 G>A) (HBB:c.93-21G>) or the most common α^0^-thalassemia deletion variants found in Greece, i.e., Mediterranean type I (NG_000006.1: g.(23641_23662)(37868_37901)del IthalID:312) and 20.5 Kb (NG_000006.1: g.(18148_18200)_(37868_37901)del IthalID:314).

## 2. Patients and Methods

### 2.1. Patients

The molecular basis of β-thalassemia variants (including Corfu δ^0^β^+^*)* was retrospectively ascertained in a total of 2558 Greek β-thalassemia heterozygotes comprising 1264 parents of 682 β-thalassemia patients followed in our Thalassemia Unit and 1294 individuals, mostly of reproductive age, referred for carrier screening between 1992 and 1998. According to the Greek thalassemia prevention program, carrier screening involves the first step of hematological phenotyping, followed by molecular genotyping driven by hematological findings consistent with β-thalassemia heterozygosity.

For the evaluation of the hematological phenotype associated with the Corfu δ^0^β^+^ allele, data from 50 heterozygotes were studied and compared to those of 58 heterozygotes with the β^+^ (IVSI-110 G>A) variant and 45 heterozygotes with α^0^ deletion variants (Mediterranean type I or 20.5Kb). For this assessment, data from children, pregnant women, subjects with iron deficiency and subjects with triplicated α or α^+^ variants were excluded.

The Ethics Committee of ‘Aghia Sophia’ Children’s Hospital approved permission for medical review, a waiver of informed consent and the anonymous publication of data, according to the 1964 declaration of Helsinki and its later amendments of October 2013. (Ethical Approval Code, 19027 02/10/2021) (www.wma.net, last access 13 December 2021)

### 2.2. Methods

Our study is a retrospective analysis of data concerning the characterization of:

(a) The hematological phenotype based on relevant red cell parameters, including Hb (g/dL), MCV (Mean Corpuscular Volume) (fl), MCH (Mean Corpuscular Hemoglobin)(pg), RDW (Red cell Distribution Width)(%), HbA_2_ (%) and HbF (%),as measured by standard hematological and biochemical methods; and
(b) The underlying genotype as evaluated with molecular methods and criteria previously described. Molecular genotyping was performed at the Laboratory of Medical Genetics (LMG), Athens University and included methods specifically applied to detect the Corfu δ^0^β^+^ variant. [6,9,10]

### 2.3. Statistical Analysis

Statistical analysis was performed utilizing Graph-Pad Prism version 8. Descriptive statistics were calculated for all phenotype variables in the two groups. For comparison of the hematological phenotype associated with heterozygotes with the Corfu δ^0^β^+^ variant, the two-tailed non-paired t test was used with the statistical significance level set at *p* = 0.05. Tukey’s box plots were used for the graphic representation of comparative measurements of RBC indices between the three groups of heterozygotes for Corfu δ^0^β^+^, α^0^ variants and IVS1-110G>A variants, respectively.

## 3. Results

### 3.1. Types and Prevalence of HBB Variants

Table 1 summarizes the types and prevalence of *HBB* variants in 2558 β-thalassemia heterozygotes; the distribution of *HBB* gene variants is listed in order of frequency. A total of 22 *HBB* gene thalassemia variants were identified: 10 null variants leading to complete impairment of β-globin synthesis (β^0^); 4 with severe reduction in β-globin synthesis (β^+^); 5 with mild (β^++^); and 3 variants with very mild (so-called silent) reduction in β-chain synthesis (β^sil^). The incidence of the “silent” variants was, in general, very low (<1%), except for 1.76% heterozygotes for the HBB:c.-151C>T (−101 C>T) variant. The most prevalent variants with an incidence of ~5% and more (considered characteristic for the Greek population) were: β^+^ IVSI-110 G>A with an incidence of 40.42%, followed by CD39 (17.67%); IVSI-1G>A (11.96%); IVSI-6 T>C (10.44%); and IVSII-745 G>A (4.93%) accounting for 85.4% (2.175/2.558) of all variants in the cohort. The incidence of the Corfu δ^0^β^+^ allele was 1.56%.

### 3.2. Corfu δ^0^β^+^ Hematological Phenotype

**Figure 1:**
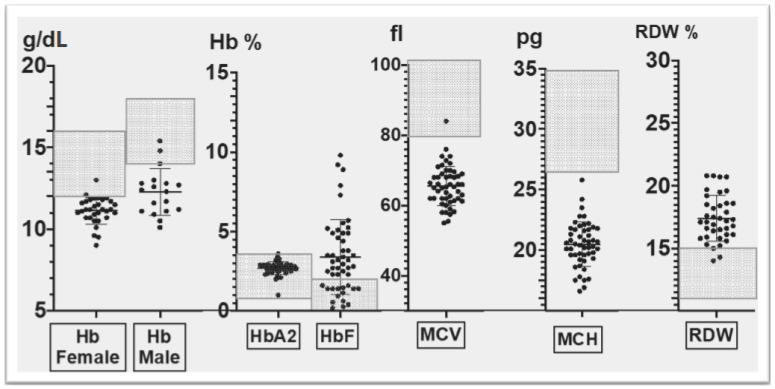
Definition of the hematological phenotype of Corfu δ^ο^β^+^; assessment of relevant hematological and biochemical parameters in 50 Corfu δ^ο^β^+^ thalassemia heterozygotes; shaded areas correspond to normal range.

To better ascertain the relevant hematological and biochemical indices of the rare Corfu δ^0^β^+^ heterozygotes, we included data from 10 additional (50 in total) heterozygotes recruited after 1998. The findings are illustrated in Figure 1. Hemoglobin levels were reduced in both male and female heterozygotes, with a mean of 12.28 ± 1.4g/dL in males and 11.13 ± 0.81 g/dL in females. Furthermore, the hematological indices of MCV, MCH and RDW were outside the normal range, such that MCV and MCH were significantly lower and RDW significantly higher in the Corfu δ^0^β^+^ thalassemia heterozygotes (Figure 1). HbA_2_ levels were within the normal range (2.7 ± 0.5 %), and HbF levels varied widely, ranging between 0.2 and 9.8% (mean: 3.39 ± 2.34%, median: 2.9%) (Figure 1).

**Figure 2:**
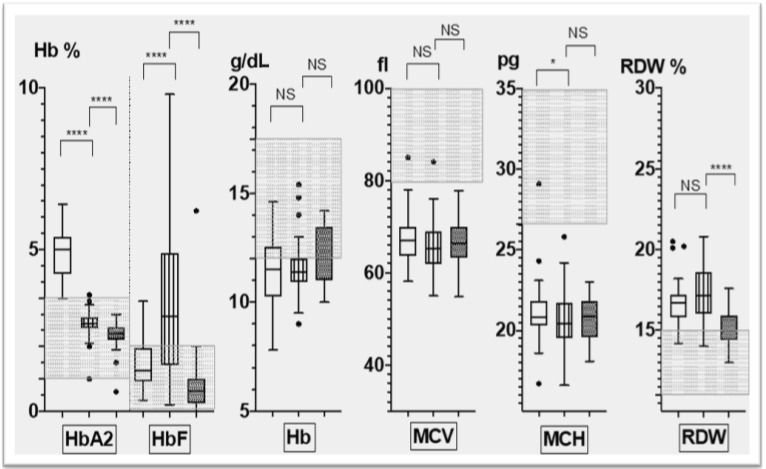
Tukey’s plots representing relevant parameters of hematological phenotypes in 50 Corfu δ^ο^β^+^ heterozygote (boxplots with lines), 58 with IVSI-110 variant (plain boxplots) and 45 with α^0^ deletion thalassemia (dotted boxplots). The upper whisker span represents the 75th percentile plus 1.5 times the interquartile distance and the lower the 25th percentile minus 1.5 times the interquartile distance. Dots represent individual values falling beyond the whiskers. Shaded areas correspond to normal range. NS: Non Significant/* *p* < 0.05. ** *p* < 0.01, *** *p* < 0.001 and **** *p* < 0.0001.

Νo significant differences in the severity of relevant red cells indices (Hb, MCV, MCH, RDW) were identified between heterozygotes with Corfu δ^0^β^+^, β^+^ (IVS1-110 G>A) or with α^0^-thalassemia heterozygotes (Figure 2). Significant differences were noted for levels of HbA_2_ and HbF. The Corfu δ^0^β^+^ heterozygotes had HbA_2_ levels in the normal range, in contrast to heterozygotes with IVS1-110 G>A, in which HbA_2_ levels were increased; and to α^0^-thalassemia heterozygotes, in which HbA_2_ levels were decreased (*p* < 0.001). Heterozygotes with Corfu δ^0^β^+^ had significantly higher HbF levels compared to those with β^+^ IVSI-110 G>A (*p* < 0.001). Compared to heterozygotes with α^0^-thalassemia deletion variants, no differences were found in the hematological phenotype of Corfu δ^0^β^+^ heterozygotes, except for the highly significant differences in HbF (*p* < 0.001) and the lower levels in HbA2 and RDW (Figure 2).

## 4. Discussion

Studies on the molecular basis and worldwide distribution of β-thalassemia variants have identified more than 300 variants with an extremely heterogeneous distribution. Most β-thalassemia variants are rare, whereas usually, only 3–5 variants account for more than 80% of β-thalassemia variants in any given population and follow a population-specific manner.

In this study, the molecular characterization of 2558 β-thalassemia heterozygotes identified 22 β-thalassemia gene variants, five of which were the most common (~85.4%). The incidence of the Corfu δ^ο^β^+^ variant allele, a unique variant so far reported exclusively in individuals of Greek origin, was 1.56%. In a previous assessment, the Corfu δ^ο^β^+^ variant was shown to account for ~36% of normal HbA2 type 2 β-thalassaemia heterozygotes [6]. The remaining heterozygotes concerned the coinheritance of either β^ο^ or β^+^ variants in trans or in cis with δ^ο^ or δ^+^ thal variants or mild β^++^ thal variants [11], the detection of which may enable differential diagnosis. It is of interest that neither of the Corfu δ^ο^β^+^ components, namely the Corfu *HBD* deletion and the *HBB* IVSI-5 G>A variant, have so far been observed independently in Greece and Cyprus, suggesting a founder effect for the compound allele [11].

In a similar study on 3769 Greek β-thalassemia heterozygotes, a total of 33 *HBB* gene variants were identified, of which the same most prevalent five variants covered 90.4% of the heterozygotes. The IVSI-5 G>A variant was detected in 14 β-thalassemia heterozygotes, whereas the δ^0^ of the Corfu δ^0^β^+^ variant was not assessed and may have escaped detection [12].

In this report, the largest group of patients with Corfu δ^0^β^+^ heterozygosity is presented. In this respect, we assessed the hematological phenotype of Corfu δ^0^β^+^ heterozygotes and compared it to β^+^-thalassemia heterozygotes with either the most common Greek variant (IVSI-110 G>A), which has similar severity to the IVSI-5 G>A β^+^ variant of the Corfu δ^0^β^+^ allele, or to heterozygotes with deletion type α^0^ thalassemia variants. As illustrated in Figure 2, hematological red cell indices were similar, whereas HbA_2_ and HbF levels differed. Corfu δ^0^β^+^ heterozygotes showed normal HbA_2_ in contrast to the significantly increased levels in IVSI-110 G>A heterozygotes and significantly lower in heterozygotes with deletion type α^0^ thalassemia. In respect to HbF, the majority of Corfu δ^0^β^+^ heterozygotes showed considerably elevated HbF levels, in most above 4%, similar to that of classical heterozygotes of the (δβ)^0^ thalassemia [6].

The clinical and hematological phenotypes of Corfu δ^0^β^+^, either homozygotes or compound heterozygotes with β^0^ thalassemia variants, have been previously presented in a very small number of patients. All patients had the clinical phenotype of non-transfusion-dependent thalassemia with moderate anemia in childhood (range of Hb 7.2–9.2g/dL), low HbA (<10%) and high levels of HbF [13]. In contrast, compound heterozygotes of other so-called “type 2 normal HbA_2_ thalassemic variants” with β^0^ or β^+^ variants have the clinical phenotype of thalassemia major, with severe anemia necessitating transfusions in the first years of life [3,9,11].

**Figure 3:**
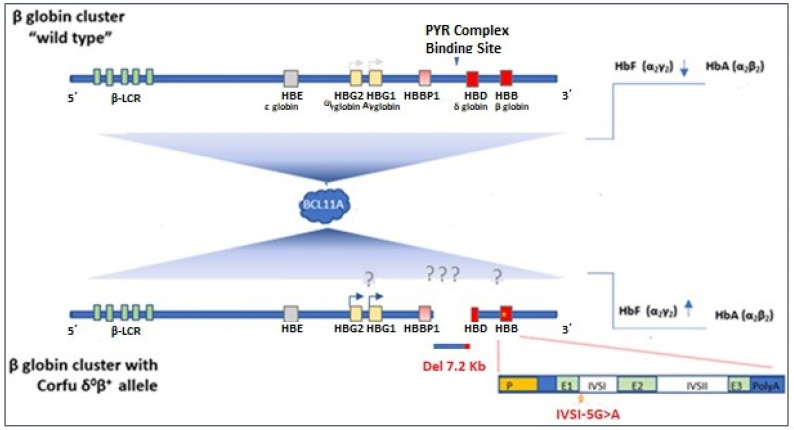
Schematic illustration of β-globin clusters (wild type and Corfu δ^0^β^+^) regarding the possible impact of BCL11A on chromatin reconfiguration and genes regulation. BCL11A has been known to enable HBG1/2 silencing through promoter interactions, although a longer-range interaction (shaded triangle) involving the entire β cluster and a region upstream HBD in specific has also been proposed [19]. In the case of 7,2Kb Corfu deletion, both this functional region and a PYR silencing complex binding site are impaired, and in the presence of the HBB:c.92+5 G>A variant, unexpected HBG1/2 gene expression (blue arrows) leads to raised HbF levels.

Gene expression studies to resolve the molecular mechanism of β-globin gene cluster regulation in the original homozygote Corfu δ^0^β^+^ patient concluded that the 7.2 kb deleted region, including the *HBD* gene, contains sequences important for the normal regulation of the *HBG1*/*HBG2, HBD* and *HBB* genes in the cluster. It appears that loss of key sequence motives in the intergenic region between *HBG1* and *HBD* are associated with disrupted (delayed) activation of the *HBB* and *HBD* genes and a concomitant increased expression of the *HBG1/HBG2* genes in cis [5,13]. Further analysis showed a 1.7kb potential repressor region upstream of *HBD* within the 7.2 kb Corfu deletion, which contains a possible binding site for the transcriptional repressor protein Bcl11a [14,15,16]. Bcl11a has been identified as a key regulator of developmental γ-globin silencing [17]. The deleted segment also contains a 250 bp sequence recognized by the chromatin remodeling PYR repressor complex, a potential regulator of hemoglobin switching, whereas chromatin conformation experiments suggested that the segment enables the locus control region of the β-globin gene cluster to activate globin expression in a developmental stage-specific manner [14] (Figure 3). Studies on primary erythroid red cell cultures measuring *HBG1/2* and *HBB* gene transcription steady state mRNA and hemoglobin expression levels in two Corfu δ^0^β^+^ homozygotes, four compound β^o^ heterozygotes and two Corfu δ^0^β^+^ heterozygotes disclosed that, in the presence of the Corfu delta deletion, a post-transcriptional mechanism disrupts *HBG1*/*2* gene silencing and potentially induces raised HbF synthesis. Thus, a combination of variants that cause a reduction in adult β-globin synthesis must be present for the Corfu delta deletion to enhance HbF production [13]. This is also supported by a report on two Italian families who carried only the Corfu *HBD* deletion variant, and in whom the 7.2 kb deleted DNA of the *HBD* gene was associated with a normal function of the “in cis” *HBB* and *HBG1*/*2* globin genes [18]. Recent studies using the CRISPR-Cas9 methodology failed to show a consistent increase in HbF and came to similar conclusions that the Corfu *HBD* deletion requires a simultaneous disruption of the ß-globin expression to lead to levels of increased γ-globin expression postnatally [14,16].

Limitations of the present study include its retrospective nature and the fact that the incidence of the allele was calculated on a partially selected population comprising parents of β thalassemia patients (cascade screening) and subjects from the general population selected for genotyping due to a hematological phenotype indicative of anemia and thalassemia heterozygosity. The cohort consisted of adults of reproductive age (preconception screening), with only a few (<20) children and pregnant women. Carrier screening included both β- and α-gene variants to allow accurate genotyping and proper genetic counseling. For the evaluation of the hematological phenotype only heterozygotes with normal ferritin and iron status were studied. Heterozygotes with any comorbidities and similar red cells changes were excluded. 

## 5. Conclusions

The Corfu δ^0^β^+^ double-variant allele accounts for a substantial proportion of β-thalassemic alleles in general and a significant proportion of thalassemic alleles with type 2 normal HbA_2_ in Greece. The milder clinical phenotype in thalassemic patients with genotypes involving the Corfu δ^0^β^+^ allele could be related to a disruption of a binding site involved in *HBG1/2* gene silencing and/or *HBB* gene activation [5,13]. Additional studies on the nature and origin of this, as well as other complex variants, are likely to provide insights into the ways that variants induce chromatin reconfiguration and enable reactivation or silencing of genes, like the β-globin cluster, such that development of respective gene-editing-based treatments may be supported. The hematological phenotype of Corfu δ^0^β^+^ heterozygotes is comparable to other β^0^ and β^+^ thalassemia heterozygotes, except the normal HbA_2_ and the significantly higher HbF levels. Even in the era of next-generation sequencing where a wider screening of alleles is applied, the hematological phenotype remains important and should be taken into account, especially in the presence of normal HbF levels, not exclusive of a Corfu δ^0^β^+^ allele in need of being distinguished from an α thalassemic variant. The precise diagnosis of Corfu δ^0^β^+^ heterozygotes is of great importance, especially in the context of genetic counseling, antenatal diagnosis and the management of patients.

## Figures and Tables

**Table 1:** Type and relative incidences of common and rare β thalassemia variants in a cohort from the Greek population of β thalassemia heterozygotes.

Ν	Thalassemia Variant		Hematologic Phenotype *	Number of Cases	Frequency (%)
	NM_000518.5	Known as			
1	c.93-21G>A	IVSI-110 G>A	β^+^	1034	40.42
2	c.118C>T	CD39 C>T	β^0^	452	17.67
3	c.92+1G>A	IVSI-1 G>A	β^0^	306	11.96
4	c.92+6T>C	IVSI-6 T>C	β^++^	267	10.44
5	c.316−106C>G	IVSII-745 C>G	β^+^	126	4.93
6	c.315+1G>A	IVSII-1 G>A	β^0^	74	2.89
7	c.-137C>G	−87 C>G	β^++^	67	2.62
8	c.20delA	Cd6 del A	β^0^	61	2.38
9	c.-151C>T	−101 C>T	β^sil^	45	1.76
10	c.92+5G>A	IVSI-5G>A plus Corfu delta	δ^0^β^+^	40	1.56
11	c.25_26delAA	Cd8 del AA	β^0^	21	0.82
12	c.17_18delCT	Cd5 del CT	β^0^	18	0.70
13	c.*6C>G	+1480 C>G	β^sil^	16	0.63
14	c.*111A>G	PolyA A>G	β^++^	14	0.55
15	c.76_92+27del	44bp del	β^0^	4	0.16
16	c.-78A>C	−28 A>C	β^++^	3	0.12
17	c.92G>C	CD30 AGG>ACG	β^0^	3	0.12
18	c.316-3C>A	IVSII-848 C>A	β^+^	2	0.08
19	c.−80T>A	−30 T>A	β^++^	2	0.08
20	c.*96T>C	+1570 T>C	β^sil^	1	0.04
21	c.114G>A	CD37 TGG>TGA	β^0^	1	0.04
22	c.135delC	CD44 del C	β^0^	1	0.04
	TOTAL			2558	100.00

* Level of suppression of β-globin synthesis: β^0^ = total; β^+^ = severe; β^++^ = mild; β^sil^ = minimal. Ν: number

## Data Availability

Data sharing is not applicable.
